# Quality Evaluation of Health Services Using the Kano Model in Two Hospitals in Peru

**DOI:** 10.3390/ijerph18116159

**Published:** 2021-06-07

**Authors:** Fernando Barrios-Ipenza, Arturo Calvo-Mora, Fernando Criado-García, Walter H. Curioso

**Affiliations:** 1 Escuela de Posgrado, Universidad Continental, Lima 15046, Peru; 2 Departamento de Administración de Empresas y Marketing, Universidad de Sevilla, 41018 Seville, Spain; schmidt@us.es (A.C.-M.); fcriado@us.es (F.C.-G.); 3 Facultad de Ciencias de la Salud, Universidad Continental, Lima 15046, Peru

**Keywords:** health services, Kano model, public–private sector partnerships, patient satisfaction, healthcare quality assessment, Peru

## Abstract

Public–private partnerships (PPP) represent an alternative model of health management focused on improving the quality of health services, particularly in emerging countries. To date, a systematic method to improve the perceived quality of health services by healthcare users in Peru has not been established. The purpose of this study was to evaluate the quality of health services in two PPP hospitals in Peru using the Kano model. A prospective cross-sectional descriptive observational study was carried out through a health service satisfaction survey using the Kano model methodology, measuring six categories of attributes. A total of 250 users of the health services were surveyed in the two PPP hospitals, located in Lima and Callao, using non-probability convenience sampling. Of the 31 attributes evaluated by the patients, 27 (81%) were classified as having a one-dimensional-type attribute, 3 (10%) were reported as mandatory, and 1 (3%) was considered as inverse. These results suggest that the presence of most of the attributes evaluated was relevant to maintaining the level of user satisfaction and that the absence of these attributes generated dissatisfaction in the users. The results showed that the users’ evaluation of health services was multidimensional—namely, their evaluation was focused not only on the interaction space between the patient and medical personnel but also addressed other interaction services.

## 1. Introduction

The quality of health services is a fundamental determinant of economic growth and development. A poor healthcare system has effects on society at the political, economic, cultural, and social levels [1]. Poor quality of health services has a direct impact on the quality of life of users, as well as on the costs of care and patient dissatisfaction [2]. On top of this, the pandemic of COVID-19 has ushered an urgent transformation of health systems and the health care quality at the global level.

Therefore, improving the quality of patient care is one of the main challenges in the health sector. Satisfied patients are more actively involved in monitoring their healthcare outcomes, completing treatments, and reducing their hospital readmissions, thus avoiding the associated costs [3,4].

From the literature on health service management models, Public–Private Partnerships (PPP) have become popular worldwide as a way of improving healthcare service delivery and are typically associated with project financing. The PPP model is based on contracts by which a public entity delegates responsibility to a private entity to manage certain health services, for which they are responsible for a specific period of time and with predetermined performance indicators [5]. In addition, PPP management models have an important component of accountability. In these management models, it is well established the responsibilities of each of the parties involved [6,7,8].

Healthcare PPPs have the potential to generate several benefits, including (i) better investment decisions, (ii) more efficient infrastructure delivery, and (iii) higher quality health services. However, PPPs are also associated with additional transaction and financing costs and may give rise to affordability challenges [7]. This model is especially suitable for emerging countries [9,10].

However, not all PPP projects will face the same set of risks. In the healthcare industry, there are risks related to demand and technology changes, as well as changing patterns in medical care [11]. With the variety of inherent risks coupled with significant relationship to the unitary charge [12], efficient risk management is, therefore, considered as challenging and demanding. However, there is evidence that the establishment of specific PPP risk management model is still low [12].

The methodologies for evaluating patient satisfaction have become a research topic in health management over recent decades. The satisfaction related to the quality of health services was initially based on the traditional one-dimensional approach, which focused only on the service received [13]. The quality assurance seminal work of Avedis Donadebian [14] identified the relevance of patient satisfaction to ensure the quality of the health services, followed by the extensive use of satisfaction evaluation focused on the interaction between the patient and the health service [15,16].

Currently, the trend of satisfaction evaluation based on a comprehensive model focuses on assessing the various components surrounding the interaction between the patient and the health service [17,18]. One of these comprehensive models is the Kano model. This model assesses the quality of care by identifying the characteristics or attributes of a service that have a high impact on patient satisfaction [19].

This change in focus is related to the evolution of the definition of quality in healthcare itself, which was initially understood as the extent to which health services for individuals and populations increases the probability of desired health outcomes, consistent with professional knowledge [20]. However, this definition does not consider the perceptions of the quality of healthcare, such as if the treatment or the information received was adequate [21].

A need to adapt the Kano model to the healthcare field arises from the lack of a systematic method to improve the quality of health services [22]. Although the Kano model was originally used to raise the quality of a product developed in the manufacturing field [23], it has also been applied in different industries and services [24], including health services [22,25,26,27] and other sectors, such as tourism [28], education [29], and banking [30].

The adaptation of this methodology represents a special challenge due to the characteristics of a health service itself, which includes intensive contact with users and the sense of timeliness of care. The use of the two-dimensional Kano model has been effective in the healthcare field because it not only indicates the quality expectations that the patient has but also allows understanding the differences in these expectations considering the characteristics of these patients [31]. In this service model, the perception of users or patients is more important than ever [32].

Only when users are adequately attended, taking into account their own perspectives of the service and associated products, can they improve and show the effectiveness of the health service used [33].

In addition, the Kano model represents a valuable opportunity to evaluate the characteristics regarding healthcare service quality improvement under the PPP model, in a comprehensive way [5,8]. Taking the above in consideration, the Kano model is presented in this paper as an alternative evaluation approach, based on the user’s perspective, and because the PPP model considers several elements, integrated to the delivery of healthcare services [22].

This study is complementary to previous research by the current authors [34], where a multidimensional scale called the HEALTHQUAL [35] was used to evaluate the quality of health services and its characteristics from the patient’s point of view. This study was conducted in two hospitals located in different geographic areas, namely, Lima and Callao in Peru. Both of our studies, [34] and the present, were carried out in the same hospitals (i.e., Barton and Kaelin), but with different samples of users.

The objectives of this study were: (1) to evaluate the quality of health services via a multidimensional approach using the Kano model methodology in the two PPP hospitals in Peru; (2) to identify and classify the attributes that define the quality of health services based on the degree of service fulfillment and the degree of customer satisfaction (i.e., must-be, one-dimensional, attractive, indifferent, and reverse); (3) to use the above classification to guide improvement actions in hospital management.

To pursue these objectives, the following sections are proposed in the article: In Section 2 the theoretical model referring to the Kano model is presented; Section 3 describes the materials and methodology that were used within the framework, according to the research objectives; the results are presented in Section 4 from the questionnaires carried out in two Peruvian hospitals; Section 5 presents the discussion of the results taking into account the objectives previously defined in Section 1, and Section 6 describes the main conclusions of the study.

## 2. Theoretical Model

Quality and excellence in service are closely related concepts, which both start from the premise of satisfying customer needs. These are two subjective terms because they depend on the perceptions and attitudes of clients regarding the services offered [36]. However, quality and excellence are not synonymous. Quality seeks to satisfy the needs of the client, while excellence goes further, attempting to generate emotions and feelings in the client [37].

As per the 2015 CEN/TS 16880 standard [38], service excellence represents the capabilities of an organization to consistently deliver exceptional customer experiences. In this sense, Asif [39] distinguished two key elements in the conceptualization of service excellence. On the one hand, the basis of the conventional concept of excellence in service is customer delight, and on the other is the need for a systematic approach to its implementation.

According to Schneider and Bowen [40], delight consists of generating emotions of pleasure and amazement in the customer that positively influence their behavior, increasing the possibility of a new purchase and promoting their loyalty. For Berman [41], the difference between customer satisfaction and delight can be understood in terms of the results of the service experience (negative or positive) and the presence of expectations (with or without prior expectations). Thus, the client will be satisfied when the result is positive in relation to their expectations. On the contrary, a customer will be dissatisfied when their expectations have not been met. However, when the client does not have a prior expectation and feels that the result of the experience is positive, delight will be achieved; otherwise, the customer will be outraged with the service.

Finally, Berman [41] indicated that customer expectations are determined by their previous experiences at the same level of service, which can lead to satisfaction simply occurring after several experiences. For this reason, excellent organizations have to strive to offer services that surprise the customer by going beyond their expectations to result in their delight.

Different approaches have addressed the problem of service excellence in the specialized literature, the most prominent of which are the Johnston model [42,43], the SERV*OR model [44], the business excellence model [45,46], and the Kano model [23].

The Johnston model challenges the traditional belief that the core of excellent service lies in exceeding customers’ expectations and delighting them. Specifically, it considers that exceeding customers’ expectations is not always feasible, as this generates an excessive use of resources and makes the service more expensive [42].

The model identifies a series of key elements in service excellence [43]:

(1) Delivering a promise: This is the central element of the model and is what makes it different to the traditional concepts of excellence in service. The organization is trustworthy, as it does what it says and does not disappoint the customer.
(2) Providing a personal touch: This makes the client feel privileged, since the organization cares about the client and offers a personalized service.
(3) Going the extra mile: This consists of dedicating extra effort, that is, going further and trying to anticipate the clients’ needs.
(4) Dealing well with problems and queries: The organization’s ability to react to a failed service is an important element for customers.

As can be seen, the Johnston model holds that organizations must act in a way that delivers what they promise, providing a personal touch (active approach), and, in the event of difficulties, adopts the appropriate measures to solve them (reactive approach) [40]. Among the limitations is the absence of a systematic approach in which structures and behaviors are established to ensure consistency in the provision of excellent services [31].

The SERV*OR model considers service orientation as a determining factor for the creation of value for the customer. Service orientation consists of the adoption of a set of policies, practices, procedures, and routines that support the behaviors that lead to achieving excellence in service [44]:

(1) Service leadership: The existence of a service leader who is in charge of motivating and helping to meet the needs of employees in their work environment is key. It is necessary to communicate a vision of service, that is, an attitude of offering quality service.
(2) Service encounters: These are the employee’s interactions with customers in the so-called “moments of truth”. Thus, how customers are treated has a direct impact on their perception of the service provided and their satisfaction. Therefore, the organization must carry out practices that create positive customer perceptions of service performance. It is also necessary to give employees the power to make decisions in this area (empowerment). In this way, staff members feel more responsible, are more motivated, and are more productive, so they respond quicker to complaints or problems that the client raises.
(3) Service systems: It is necessary to develop an integrated system of practices and procedures to achieve the provision of a quality service. This system must include practices for preventing and recovering from service failures and for communication with the customer.
(4) Human resources management: The organization must develop training measures, as the training and reward of workers are related to improvements in the provision of customer service.

For Asif [39], the most significant contribution of this model was the description of the structures that provides a systematic approach to learning and continuous improvement in service provision. However, this does not address the key aspects of service excellence, such as customer delight.

At the end of the 1980s, Business Excellence Models (BEMs) were developed. The most widespread BEMs are the Model of the European Foundation for Quality Management (EFQM) [45] and the Malcolm Baldrige National Quality Award (MBNQA) [47]. The fundamental premise of these models is that excellent results, with respect to clients and other stakeholders, are achieved through management centered on leadership, strategy, people, resources, and cooperation, as well as through having a focus on processes. In addition, BEMs are oriented toward continuous improvement, indicating that activities, such as innovation, learning, or creativity, promote and enhance the impact that management has on the results of the organization [48]. However, as Asif and Gouthier [46] indicated, BEMs are generic in nature and do not address the central elements of service excellence. They are non-prescriptive frameworks and do not provide specific guidance for their implementation at the operational level.

Health services have unique characteristics compared to other types of services. Service quality in the health sector is more important compared to that of other sectors because high-quality health services have a significant impact on health and well-being of individuals [49]. When we refer to health services, we focus on addressing and solving the physical, psychological, and social needs that people have. Therefore, it is necessary to resort to evaluation tools that collect this multidimensionality of needs [50].

Moreover, in the healthcare sector, the implementation of quality management strategies represents a critical factor for achieving excellence in health-related organizations [2,3,34].

The Kano model [23] is the framework that best adjusts to the objectives of this study, due to the limitations that the previous models present when identifying and classifying the attributes that define satisfaction, dissatisfaction, and customer delight. Likewise, due to the dimensions addressed by the Kano model, it bears a strong relationship with the intrinsic characteristics of the PPP health service management models [9]

In this way, the Kano model assumes that it is possible that the factors that influence satisfaction present a non-linear behavior; that the factors that produce satisfaction are not the same as those that produce dissatisfaction; and that there may be factors, not expected by the customer, that increase customer satisfaction more than proportionally (e.g., attributes of delight).

Traditionally, user satisfaction is measured in a one-dimensional way [51], that is, the level of satisfaction of a user is considered proportional only to the performance of the service offered [52]. However, this linear relationship between the variables of satisfaction and quality of service is imprecise [25]. To overcome this one-dimensional limitation, the Kano model provides a dualistic approach.

The Kano model classifies the quality attributes based on the degree of service compliance (horizontal axis) and the degree of customer satisfaction (vertical axis) and divides the quality attributes of products or services into five categories: (1) The mandatory or “must-be” (M), (2) attractive (A), (3) one-dimensional (O), (4) reverse (R), and (5) indifferent (I). The definition of each of the characteristics is as follows:

- Mandatory or “must-be” (M): This characteristic is a basic requirement, so its absence leads to extreme customer dissatisfaction. The customer takes this requirement for granted; therefore, its fulfillment does not increase their level of satisfaction. Compliance with this requirement leads to a status of “satisfied”.
- One-dimensional (O): This characteristic is a linear-type requirement; when it is met, customer satisfaction increases, but when it is not met, the level of customer satisfaction decreases, that is, their dissatisfaction increases. This characteristic is what customers expect from the proposed service; it is a performance requirement that is typically demanded by customers.
- Attractive (A): This feature has the greatest impact on the level of customer satisfaction. The client may not express or expect this feature explicitly; however, its presence increases satisfaction more than proportionally. Moreover, if not met, satisfaction does not diminish. Consequently, attractive requirements make it possible to differentiate the product/service from the competition. This feature comprises the attributes related to customer delight.
- Indifferent (I): This characteristic presents a requirement of no preference, which implies that the customer is indifferent to the characteristic.
- Reverse (R): This characteristic is one that creates customer satisfaction when absent and dissatisfaction when it occurs.

**Figure 1:**
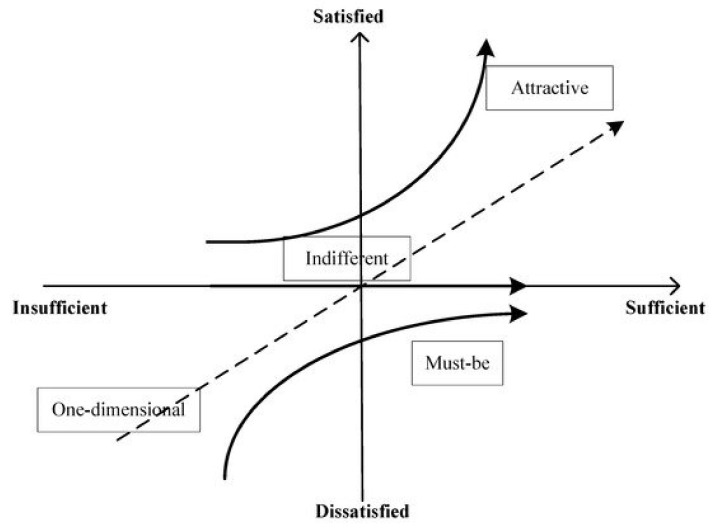
The Kano model [23], as suggested by Jin et al. [53].

The Kano model, as suggested by Jin et al. [53], is presented in Figure 1.

## 3. Materials and Methods

### 3.1. Participants

A prospective cross-sectional descriptive observational study was carried out through a health service satisfaction survey using the Kano model methodology. A non-probabilistic sampling was carried out to select a sample of 250 users who attended outpatient services in the two PPP hospitals in Lima and Callao. Both hospitals include primary healthcare services and are located nearby, and both have been operating for 11 years and are part of the Social Security system. Users were purposively selected from the outpatient services by the interviewers. Both hospitals serve more than 250,000 patients annually. The information was collected in January 2019. A total of 128 users were evaluated at the Guillermo Kaelin de la Fuente Hospital (Lima) and 122 users at the Alberto Barton Thompson Hospital (Callao).

The process of disseminating this questionnaire was based on face-to-face interviews. For this, those in charge of collecting information approached the users once the users had finished with their health service in the selected hospitals. Previously, we asked permission from the PPP managers at both hospitals for the application of the questionnaires.

The study was conducted in agreement with the Declaration of Helsinki. The researchers only had access to anonymized data. Since the study used only secondary data retrieved, no ethics approval was obtained. Conventions of good scientific practice, data protection and information security have been applied in analyzing the data and presenting the results in the following sections.

### 3.2. Instrument

For the construction of the instrument, we adapted the dimensions and attributes of our previous work [34] that used the multidimensional HEALTHQUAL scale, which measures the quality of health services and assesses the characteristics from a patient’s perspective. Thus, the dimensions and attributes evaluated were: (1) health personnel; (2) non-health personnel; (3) installations, equipment, and tangible elements; (4) efficiency. The HEALTHQUAL scale has been validated previously in various contexts to assess the quality of health services [2,34,35].

For the identification of the attributes that define a service and their classification, the Kano model uses a structured questionnaire that contains two types of questions for each attribute. On the one hand, there are functional questions, which ask how the customer feels if the attribute is present in the service; on the other, there are dysfunctional questions, which ask how the client feels when the attribute is not present in the service [23,54].

The Kano model uses pairs of functional and dysfunctional questions for each service attribute; the functional attribute examines whether there is a present or adequate level of quality, while the dysfunctional attribute assumes that the quality is inadequate or insufficient. The alternative responses of the instrument questions were presented on a five-point Likert scale [23]. The alternative responses were: (1) I like it, (2) I hope it is so, (3) I am neutral, (4) I can accept it being that way, (5) I dislike it that way. In addition, the instrument included questions pertaining to sex, age, and frequency of use of health services.

A total of 65 questions were included in the questionnaire. Three of these questions correspond to demographic questions such as sex, age and the hospital where they were treated; the remaining 62 questions are related to Kano model shown in Table 1.

### 3.3. Methods and Data Analysis

The user perceptions were classified into six categories. In this study, in addition to the five original categories of the Kano model [23], one more linked to the attribute, called questionable (Q), was incorporated, which suggests that it is not clear whether the quality is the one expected by the user, and was originally reported by other authors in Greece and Indonesia [55,56]. From the information reported, the responses of the interviewees were classified as (Table 2): Must-be attributes (M), one-dimensional attributes (O), attractive attributes (A), indifferent attributes (I), questionable attributes (Q), and reverse attributes (R); this table presents the process for identifying attributes based on the questions and the type of response provided by the participants.

Based on previous studies about the Kano model methodology, we carried out a descriptive univariate analysis. Therefore, the coefficients of satisfaction (CS) and dissatisfaction (DS) were calculated using the methodology proposed by Berger, Blauth and Boger [57], which are widely used in the health-related Kano model literature [22,25,55]. These coefficients can be used to find the average impact of a requirement on customer satisfaction. To calculate the average impact on customer satisfaction, the attributes one-dimensional and attractive were added and divided by the total sum of the attributes attractive, must-be, one-dimensional, and indifferent. To calculate the impact of dissatisfaction, it was necessary to add the must-be and one-dimensional columns and to divide it by the same factor as in the case of satisfaction (Table 2).

These coefficients have a maximum value of CS = 1 and DS = −1. Thus, CS indicates how much the customer satisfaction increases if provided an “attractive” or “one-dimensional” type of attribute (if better than that of the competition, the score increases by this coefficient). On the contrary, DS indicates how much customer satisfaction decreases if the attribute is not provided, in this case, “mandatory” or “one-dimensional” (if worse than the competition, the score decreases in this coefficient).

#### Kano Questionnaire Reliability

The item analysis was performed to evaluate the internal reliability of the Kano questionnaire. Cronbach’s alpha is a measure of the internal consistency of an instrument (i.e., questionnaire) and expressed as a number between 0 and 1. A Cronbach’s alpha value of 0.7 is generally considered to be an acceptable reliability coefficient, as Materla et. al. previously stated [25]. Based on the above criteria, our Kano questionnaire showed an acceptable internal consistency with a Standardized Cronbach’s alpha value of 0.75.

## 4. Results

Considering the demographic characteristics of the participants (Table 3), the highest percentage of participants is related to 35 to 54 years old (40%). Regarding gender, the percentage of women is 51% (128). When it comes to hospital facility, 51% of respondents (128) were from Barton Thompson.

The results were analyzed by the dimensions of the HEALTHQUAL scale considering the classification obtained for the attribute, as well as the level of satisfaction and dissatisfaction reported.

### 4.1. Health Personnel (PS)

For this dimension, the results of the evaluated attributes were one-dimensional. This indicates that satisfaction occurs when they are fulfilled and dissatisfaction when they are not fulfilled.

These positive characteristics are related to customer satisfaction—that is, the more the characteristics are met, the higher the degree of customer satisfaction, and vice versa. For example, the more personalized the service offered by health personnel, the more satisfied the user is. They represent performance needs (one-dimensional quality).

Regarding the degree of satisfaction or dissatisfaction, we observed that for all of the functional questions related to health personnel, they were linked to a higher level of dissatisfaction rather than satisfaction with the presence of said characteristic. For example, the question about the friendliness and courtesy of health personnel presented a DS coefficient of −0.94, which led to proportionally greater dissatisfaction in the absence of these characteristics in relation to service satisfaction, compared with a CS of 0.69 that proportionally increased the satisfaction with the service to a lesser extent when these characteristics (kindness and courtesy) were present.

Table 4 presents the classification for each one of the functional questions corresponding to the health personnel dimension, as well as the scores for the degree of satisfaction and dissatisfaction for each of the elements.

**Figure 2:**
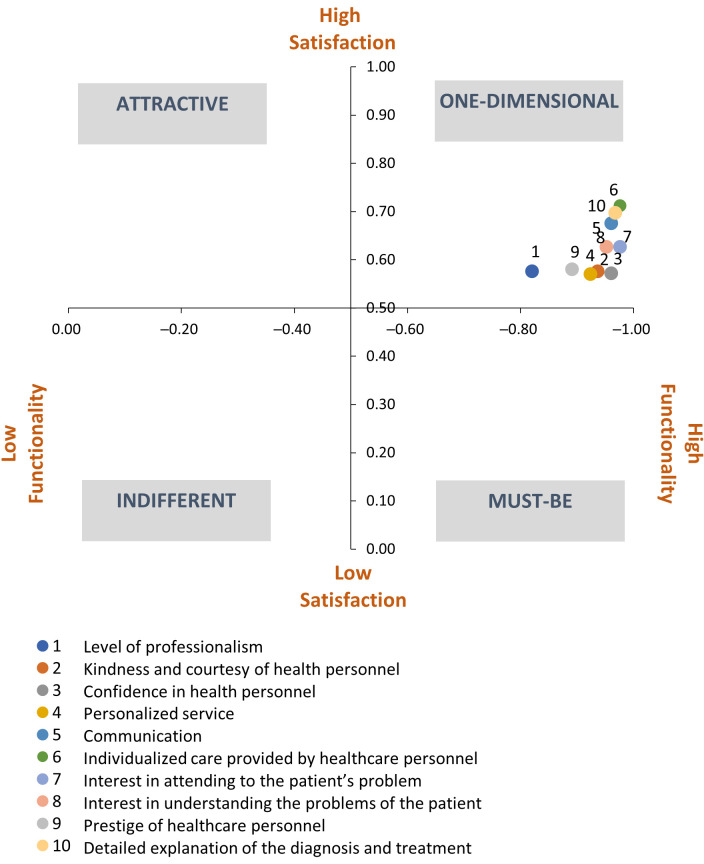
Classification of the attributes of the health personnel dimension.

Although the functional questions were associated with one-dimensionality, some of the characteristics of this domain (i.e., professionalism, kindness and courtesy, trust, personalized service, and prestigious staff) were found to be near the mandatory section (Figure 2).

### 4.2. Non-Health Personnel (PNS)

As per the previous dimension, the functional questions corresponding to the non-health personnel dimension were one-dimensional. Table 5 presents the results at the classification level of the functional questions, as well as the levels of satisfaction and dissatisfaction reported.

In relation to these last two indicators, the same trend was observed as in the health personnel, that is, the existence of these characteristics evaluated in the functional questions did not generate such a high level of satisfaction compared to the dissatisfaction that users experienced in the cases where such qualities did not exist. In the three functional questions, the score obtained was −0.97.

**Figure 3:**
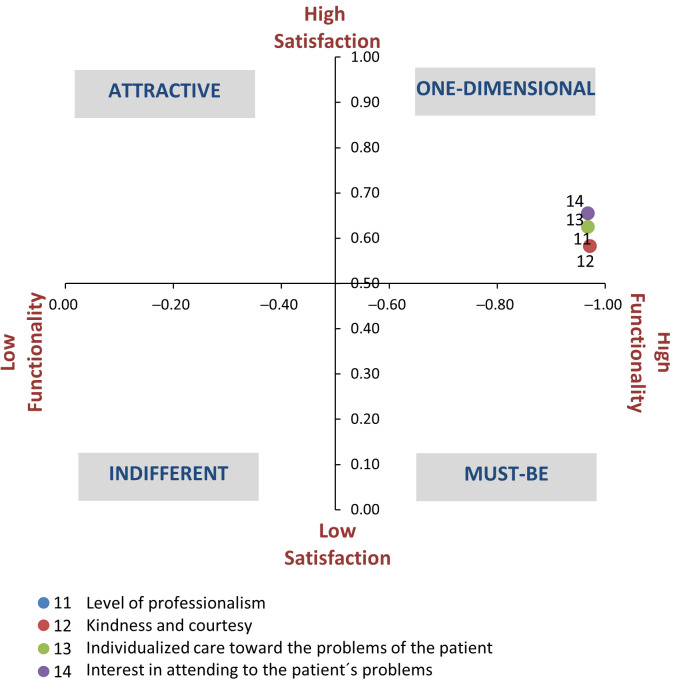
Classification of the attributes of the non-healthcare personnel dimension.

Figure 3 shows that the attributes “professionalism” and “kindness and courtesy” were close to the mandatory quadrant.

### 4.3. Facilities, Equipment, and Tangibles (IEET)

The classification of the attributes referring to the facilities, equipment, and tangibles (Table 6) indicates that these were considered one-dimensional. Regarding the degree of satisfaction and dissatisfaction, we observed that, as in the other dimensions, the dissatisfaction indicator, in absolute terms, was higher compared to the satisfaction indicator.

However, the difference between both indicators was lower compared to the results obtained in the other dimensions. Considering that the maximum score that can be obtained in the indicator is 1 for the satisfaction indicator and −1 for the dissatisfaction indicator; in all of the functional questions, the score was very close to this indicator. This means that the existence or non-existence of each of these characteristics greatly influences the satisfaction experienced by the service by users.

**Figure 4:**
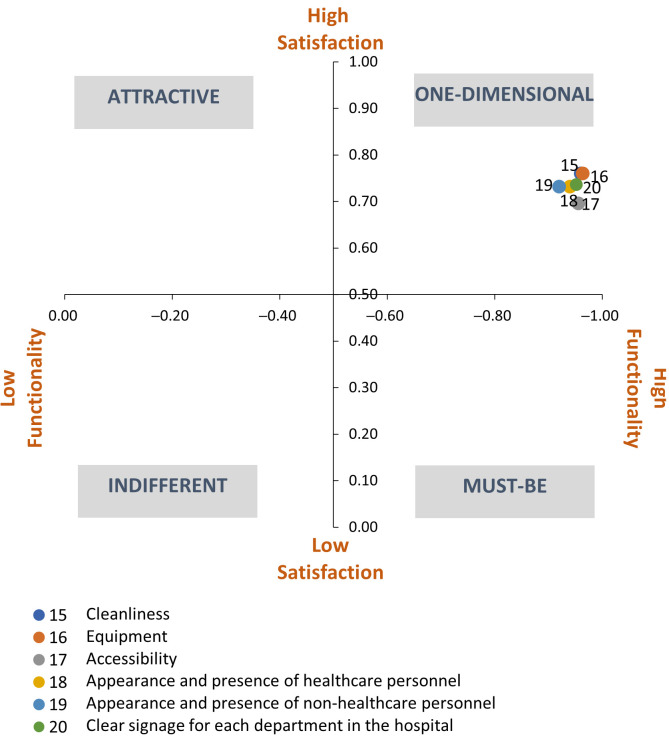
Classification of the attributes of the facilities, equipment, and tangibles dimension.

According to the model, the evaluated attributes were considered by users as exclusively one-dimensional. Figure 4 presents this distribution of the indicators in the one-dimensional quadrant.

### 4.4. Efficiency (E)

The classification of the functional questions related to the efficiency of the service indicators was primarily one-dimensional, except for the attributes related to “the possibility of making a medical appointment,” “the level of bureaucracy,” and the “resolution of complaints,” which were classified as mandatory. These features that address functional questions, while not increasing customer satisfaction, cause strong dissatisfaction if they are not present. They represent basic or expected needs (basic or required quality). In addition, we identified an attribute as inverse: the “side effects of medicines” (Table 7).

This classification is related to the scores obtained in the satisfaction and dissatisfaction indicators. In the case of the functional questions linked to the mandatory classification, the reported CS was less than 0.5 and the SD was close to −1.

**Figure 5:**
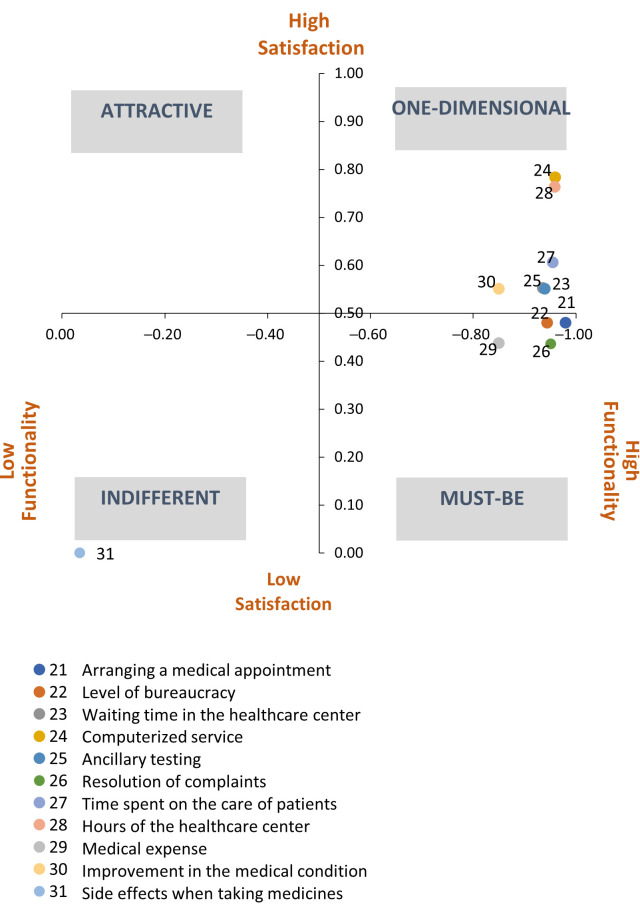
Classification of the attributes of the efficiency dimension.

Based on a visual interpretation of the Figure 5, for the one-dimensional functional questions, the dissatisfaction indicator was higher than the satisfaction indicator. Regarding the side effects attribute, only in the case of the occurrence related to the consumption of a medicine was classified as reverse in relation to the satisfaction experienced.

## 5. Discussion

The first objective of the study was to evaluate the quality of health services through a multidimensional approach using the Kano model in two Peruvian hospitals. To our knowledge, this is the first published article in Peru that have explored the Kano model and its relationship with the healthcare sector. In this sense, this is a pioneering study and contributes to closing the existing gap in the literature on this topic. When it comes to the second and third objectives, we found that one-dimensional attributes were typically considered basic and indispensable regarding the provision of healthcare services [29,58] unlike attributes such as “attractive” and “must-be”, which require a higher level of understanding for healthcare services users. Therefore, the focus must be prioritized on one-dimensional attributes to enhance patient satisfaction, attract new customers, and gain patient loyalty. One of the reasons regarding why few attributes were reported in the “must-be” efficiency dimension may be because the two Peruvian hospitals provide primary healthcare services which usually do not require prolonged treatment regimens [25].

Regarding CS and DS coefficients reported, due to the unidimensional nature of the majority of the functional questions, the scores were higher than 0.5 in the case of CS and −0.5 in the case of DS. These indicators suggest that, in the presence of the characteristics exposed in the functional questions, the level of satisfaction increased; on the contrary, their absence generated a high level of dissatisfaction in users. In terms of absolute values, a higher score is observed for the case of DS compared to CS, even many of these indicators are close to 1.

Moreover, focused on the third objective regarding the use of the above classification to improve hospital management and health care quality, the lack of resources in Peruvian hospitals (as in many Latin American countries) constitutes a great challenge that requires not only a significant budget increase but also demand an effective management of resources. On top of that, there is an insufficient number of health professionals, and many of them are inadequate trained in urban and rural areas in Peru [59]. Finally, besides legislation for PPPs was published in 2008 regarding infrastructure, maintenance, and service provisions; it was only until 2013 when PPP began to be implemented for health services [59]. Therefore, the implementation of PPPs in the Peruvian health sector represents an opportunity to promote and guarantee the quality of health services, mainly among users excluded from the system [59,60]. The results presented are commonly found in other healthcare facilities in Peru [59] that operate under a modality similar to that of the hospitals evaluated in this study. From this perspective, hospital management under PPP is more prone to implement service quality standards to ensure users’ satisfaction as in other countries, such as Malaysia [61,62], Turkey [63], and India [64].

A multidimensional approach to identify healthcare needs used in the two Peruvian hospitals not only provides specific information on how to use the Kano model for a specific healthcare facility to identify patient needs, but also this study validates the reliability of the Kano survey in the two Peruvian hospitals using the Cronbach’s alpha [25].

## 6. Conclusions

In the health sector, maintaining and preserving a high level of satisfaction is a permanent challenge for health operators due to the multidimensionality of the attributes that these services possess [34]. Likewise, the findings of this study provide information on which characteristics of the health services should prevail and be promoted to generate a higher level of satisfaction, particularly in a reality, such as Peru, where the levels of satisfaction with health have been low and focused on the relationship between the patient and the doctor [58,59,60,61].

User’s satisfaction in health services goes beyond caring for just the patient. It is important to provide individual attention to each healthcare domain in order to effectively meet patient needs. Therefore, instruments such as those used in this study (based on the Kano method and the HEALTHQUAL scale [34]) would help to better understand not only the perceptions of users, but also to differentiate healthcare services and hospitals from competitors [25]. The Kano model can be a useful tool for healthcare managers to determine which attributes have the chance to increase or decrease satisfaction and can be applied to efficiently use the available resources by focusing only on the attributes that have a high impact on satisfaction in events such as the current COVID-19 pandemic, natural disasters, and economic recession, among others [25].

In this sense, the use of more than one instrument to evaluate the quality of health services would allow health managers to better understand the various quality aspects of the patient´s treatment cycle and the service lifecycle to increase healthcare service quality and enhance patient satisfaction [25]. Thus, this evaluation represents an opportunity to improve the quality of care in health services and is useful for decision-makers working to improve the management of health services [65]. Healthcare managers and decision makers must capture changing patient needs over time to continuously improve the healthcare service quality using the Kano model. Involving patients in the decision-making process and treatment decisions can give valuable insights into varying patient expectations due to increased patient awareness and empowerment [19,25]. Moreover, the Kano model might assist healthcare providers in eliciting patient preferences in the development of healthcare services and in the quality improvement process [25,55].

The integration of different methodologies to evaluate health services has been used previously by researchers such as Wongrukmit and Thawesaengskulthai [66] and Sulisworo [56], since it allows a greater understanding of how patients evaluate these services. In this case, we applied the Kano model to classify attributes based on the ratios of satisfaction/dissatisfaction and based on previous reports from the literature related to hospital services. Such classification provides a good framework to managers to better allocate their budgets and resources for improving healthcare services quality management programs.

As most attributes are one-dimensional, this represents an opportunity to ensure that these attributes are kept as a direct reference to the user preferences.

In the case of mandatory attributes, these represent a great challenge because improvements in these attributes do not generate greater satisfaction; however, their absence leads to a significant level of dissatisfaction. Therefore, we can suggest to the quality management offices of both PPP hospitals, to include the Kano questionnaire as part of the quality performance indicators in their annual quality plans. Furthermore, in the context of the COVID-19 pandemic, the approach applied in this research could be replicated in other hospitals in the Latin America region toward quality management within the healthcare industry.

However, this study has certain limitations. Although it was conducted in two hospitals in Peru, the results and interpretation should be handled carefully when extrapolating the findings to other populations with different sociodemographic, cultural, infrastructure, and hospital management characteristics. Other classification methodologies (e.g., using a different number of attribute categories) may provide different results.

Likewise, it would be interesting if future studies not only incorporated a greater number of hospitals to analyze the consistency of the results but also compared the levels of satisfaction reported, including other types of hospital management beyond PPPs. Future research should consider other user satisfaction instruments to evaluate the consistency of the results. For example, due to the prominence of the “one-dimensional” attribute over other attributes, adding categories such as “high level of one-dimensional” or “slightly one-dimensional” could be included to perform a more detailed attribute categorization, as suggested by Kano et al. [23]. Moreover, in the context of the COVID-19 pandemic, online instruments should be properly discussed and evaluated considering the socioeconomic factors and broadband access [67] in rural and urban areas in Peru.

In conclusion, our results, based in the two Peruvian hospitals (Barton and Kaelin), showed that the user´s evaluation of health services was multidimensional—namely, their evaluation was focused not only on the interaction space between the patient and medical personnel but also addressed other interaction services.

## Figures and Tables

**Table 1:** Description of the dimensions and attributes evaluated in this study.

Dimension	Service Attribute
Health personnel	1. Level of professionalism
	2. Kindness and courtesy of health personnel
	3. Confidence in health personnel
	4. Personalized service
	5. Communication
	6. Individualized care provided by healthcare personnel
	7. Interest in attending to the patient’s problem
	8. Interest in understanding the problems of the patient
	9. Prestige of healthcare personnel
	10. Detailed explanation of the diagnosis and treatment
Non-health personnel	11. Level of professionalism
	12. Kindness and courtesy
	13. Individualized care toward the problems of the patient
	14. Interest in attending to the patient´s problems
Facilities, equipment, and tangibles	15. Cleanliness
	16. Equipment
	17. Accessibility
	18. Appearance and presence of healthcare personnel
	19. Appearance and presence of non-healthcare personnel
	20. Clear signage for each department in the hospital
Efficiency	21. Arranging a medical appointment
	22. Level of bureaucracy
	23. Waiting time in the healthcare center
	24. Computerized service
	25. Ancillary testing
	26. Resolution of complaints
	27. Time spent on the care of patients
	28. Hours of the healthcare center
	29. Medical expense
	30. Improvement in the medical condition
	31. Side effects when taking medicines

**Table 2:** Calculation of the degree of satisfaction and dissatisfaction using the Kano model.

Features	Calculation
Degree of satisfaction (CS)	Attractive + one-dimensional Attractive + one-dimensional + mandatory + indifferent
Degree of dissatisfaction (DS)	Mandatory + one-dimensional Attractive + one-dimensional + mandatory + indifferent

**Table 3:** Descriptive analysis regarding demographic characteristics of the sample.

Variables		*n*	%
Age	18–34	67	27%
	35–54	101	40%
	55–74	60	24%
	75+	22	9%
Gender	Male	122	49%
	Female	128	51%
Hospital	Barton Thomson	128	51%
	Guillermo Kaelin	122	49%

**Table 4:** Results corresponding to the health personnel dimension.

Question	Attributes	Quality Levels ^1^						Classif.	CS ^2^	DS ^3^
		A	Q	I	R	M	O			
1	How do you feel about the level of professionalism of the healthcare personnel?	24	0	21	0	85	120	O	0.58	−0.82
2	If the healthcare staff are kind and courteous, how do you feel?	12	0	4	0	74	160	O	0.69	−0.94
3	If the healthcare staff are confident, how do you feel?	2	0	8	0	99	141	O	0.57	−0.96
4	If the health personnel provide a personalized service, how do you feel?	10	1	9	0	98	132	O	0.57	−0.92
5	How do you feel if the communication with the healthcare personnel is good?	7	0	3	0	78	162	O	0.68	−0.96
6	How do you feel if the individualized care of the healthcare personnel is good?	4	0	2	0	70	174	O	0.71	−0.98
7	How do you feel if the healthcare personnel show interest in solving your problems?	4	1	2	0	91	152	O	0.63	−0.98
8	How do you feel if the health staff show interest in understanding your problems?	8	0	4	1	89	148	O	0.63	−0.95
9	If the medical staff are prestigious, how do you feel?	17	0	10	2	94	127	O	0.58	−0.89
10	If doctors explain in detail the diagnoses and treatment of a disease, how do you feel?	5	0	3	2	72	168	O	0.70	−0.97

^1^ A = Attractive, I = Indifferent, M = Must-be, O = One dimensional, Q = Questionable, and R = Reverse. ^2^ Degree of satisfaction (CS). ^3^ Degree of dissatisfaction (DS).

**Table 5:** Results corresponding to the non-healthcare personnel dimension.

Question	Attributes	Quality Levels ^1^						Classif.	CS ^2^	DS ^3^
		A	Q	I	R	M	O			
11	If non-healthcare personnel act professionally, how do you feel?	1	0	6	1	98	144	O	0.58	−0.97
12	If non-healthcare staff are kind and courteous, how do you feel?	5	0	2	1	75	167	O	0.69	−0.97
13	How do you feel if non-healthcare personnel attend to your problems?	3	0	5	2	88	152	O	0.63	−0.97
14	How do you feel if non-healthcare personnel show interest in solving your problems?	8	0	0	1	86	155	O	0.65	−0.97

^1^ A = Attractive, I = Indifferent, M = Must-be, O = One dimensional, Q = Questionable, and R = Reverse. ^2^ Degree of satisfaction (CS). ^3^ Degree of dissatisfaction (DS).

**Table 6:** Results corresponding to the facilities, equipment, and tangibles dimension.

Question	Attributes	Quality Levels ^1^						Classif.	CS ^2^	DS ^3^
		A	Q	I	R	M	O			
15	How do you feel about the cleanliness of the facilities?	6	0	4	0	56	184	O	0.76	−0.96
16	If the healthcare center equipment is adequate, how do you feel?	8	0	1	0	81	160	O	0.67	−0.96
17	If the accessibility to the healthcare center is adequate, how do you feel?	7	0	4	0	72	167	O	0.70	−0.96
18	If the appearance and presence of the healthcare personnel are adequate, how do you feel?	10	0	5	0	62	173	O	0.73	−0.94
19	If the appearance and presence of non-healthcare personnel are adequate, how do you feel?	11	0	9	0	58	172	O	0.73	−0.92
20	Given the existence of clear signage for each department in the hospital, how do you feel?	9	0	3	0	63	175	O	0.74	−0.95

^1^ A = Attractive, I = Indifferent, M = Must-be, O = One dimensional, Q = Questionable, and R = Reverse. ^2^ Degree of satisfaction (CS). ^3^ Degree of dissatisfaction (DS).

**Table 7:** Results corresponding to the efficiency dimension.

Question	Attributes	Quality Levels ^1^						Classif.	CS ^2^	DS ^3^
		A	Q	I	R	M	O			
21	Given the many facilities to arrange a medical appointment, how do you feel?	2	0	3	0	127	118	M	0.48	−0.98
22	If the level of bureaucracy is low, how do you feel?	3	0	11	0	122	114	M	0.47	−0.94
23	Given the adequate waiting time before entering a medical consultation, how do you feel?	2	0	14	0	98	136	O	0.55	−0.94
24	If the hospital provides a very good computerized service, how do you feel?	8	0	2	1	52	187	O	0.78	−0.96
25	Given the adequate speed of ancillary testing, how do you feel?	5	1	10	2	101	131	O	0.55	−0.94
26	Faced with the proper resolution of complaints, how do you feel?	3	0	9	2	131	105	M	0.44	−0.95
27	Given the adequate time of care of each patient, how do you feel?	4	1	7	3	90	145	O	0.61	−0.96
28	Given the appropriate hours of attention at the healthcare center, how do you feel?	5	0	5	5	53	182	O	0.76	−0.96
29	Given the adequate medical expenses made by the patient, how do you feel?	2	0	35	3	104	106	O	0.44	−0.85
30	If there is an improvement in the medical condition as a result of the efforts and treatment by medical personnel, how do you feel?	14	1	23	2	88	122	O	0.55	−0.85
31	Given the occurrence of side effects when taking medicines, how do you feel?	0	1	28	220	1	0	R	0.00	−0.03

^1^ A = Attractive, I = Indifferent, M = Must-be, O = One dimensional, Q = Questionable, and R = Reverse. ^2^ Degree of satisfaction (CS). ^3^ Degree of dissatisfaction (DS).

## Data Availability

The data presented in this study are available on request from the corresponding authors.
